# Nanoparticles from culture media are internalized by *in vitro*-produced bovine embryos and its depletion affect expression of pluripotency genes

**DOI:** 10.1590/1984-3143-AR2020-0028

**Published:** 2021-05-10

**Authors:** Bárbara Melo-Báez, Edwin A. Mellisho, Joel Cabezas, Alejandra E. Velásquez, Daniel Veraguas, Diego Andrés Caamaño Escobar, Fidel O. Castro, Lleretny Rodríguez-Álvarez

**Affiliations:** 1 Laboratorio de Biotecnología Animal, Facultad de Ciencias Veterinarias, Universidad de Concepción, Chillán, Chile; 2 Centro de investigación en Tecnología de Embriones, Facultad de Zootecnia, Universidad Nacional Agraria La Molina, Lima, Perú

**Keywords:** EVs, nanoparticles, culture medium, bovine embryos, pluripotency genes

## Abstract

Extracellular vesicles are nanoparticles secreted by cell and have been proposed as suitable markers to identify competent embryos produced *in vitro*. Characterizing EVs secreted by individual embryos is challenging because culture medium itself contributes to the pool of nanoparticles that are co-isolated. To avoid this, culture medium must be depleted of nanoparticles that are present in natural protein source. The aim of this study was to evaluate if the culture medium subjected to nanoparticle depletion can support the proper *in vitro* development of bovine embryos. Zygotes were cultured in groups on depleted or control medium for 8 days. Nanoparticles from the medium were characterized by their morphology, size and expression of EVs surface markers. Isolated nanoparticles were labelled and added to depleted medium containing embryos at different developmental stages and evaluated after 24 hours at 2, 8-16 cells, morula and blastocyst stages. There were no statistical differences on blastocyst rate at day 7 and 8, total cell count neither blastocyst diameter between groups. However, morphological quality was better in blastocysts cultured in non-depleted medium and the expression of SOX2 was significantly lower whereas NANOG expression was significantly higher. Few nanoparticles from medium had a typical morphology of EVs but were positive to specific surface markers. Punctuated green fluorescence near the nuclei of embryonic cells was observed in embryos from all developmental stages. In summary, nanoparticles from culture medium are internalized by *in vitro* cultured bovine embryos and their depletion affects the capacity of medium to support the proper embryo development.

## Introduction

*In vitro* produced (IVP) embryos can be transferred to a maternal recipient in the one-cell stage; however, in most species, embryos are usually cultured for several days before transfer, to reach a more advanced developmental stage (e.g. blastocyst). *In vivo*, after fertilization, bovine embryos move through the oviduct reaching the uterus at the stage of 8-16 cells (Hackett et al., 1993) whereas IVP embryos need to be exposed to artificial conditions that mimic the natural milieu provided by the oviduct. *In vitro* environment alters embryonic developmental kinetics, gene expression pattern, cell signaling, metabolism and chromosome structure among others (Wrenzycki et al., 2001; Rizos et al., 2003; Rodríguez-Alvarez et al., 2010; Cagnone and Sirard, 2016). Those alterations contribute to the low competence of IVP embryos, leading to high embryonic loss before embryo-maternal recognition, as well as to long term effects such as pregnancy loss and lower birth rates (Thatcher et al., 2001; Diskin and Morris, 2008; Berg et al., 2010; Hansen et al., 2010; Besenfelder et al., 2012; Perkel et al., 2015).

Despite all the efforts, *in vitro* systems cannot reproduce the optimal conditions supplied by the maternal environment. The reciprocal interactions between the oviduct cells and the embryo, set the bases for normal embryonic development and prepares the maternal side for pregnancy recognition and implantation (Lee et al., 2002; Almiñana et al., 2012; Maillo et al., 2015; Smits et al., 2016). Absence of embryo-maternal signaling during the transit of the embryo through the oviduct in early development, may explain in part the low success of IVP technologies. The mechanisms involved in embryo-maternal crosstalk in the oviduct are still unknown (Fazeli, 2008). Recently, it has been demonstrated that both the oviduct cells and pre-implantation embryos, secrete extracellular vesicles (EVs), including microvesicles and exosomes, as a mechanism of embryo-maternal communication (Valadi et al., 2007; Ng et al., 2013; Al-Dossary et al., 2013; Saadeldin et al., 2014; Burns et al., 2014; Lopera-Vásquez et al., 2016; Giacomini et al., 2017; Almiñana et al., 2017; Szekeres-Bartho et al., 2018; Ávila and Silveira., 2019; Almiñana and Bauersachs., 2020). EVs are nanoparticles secreted by different cell types and act as mediators of short and long-distance signals by their cargoes such as proteins, lipids, mRNA and microRNAs, that are internalized by target cells (Théry et al., 2009; Mathivanan et al., 2010).

The role of EVs during early embryo development and its communication with the maternal environment has been the subject of recent studies (Almiñana et al., 2012, 2017, 2018; Szekeres-Bartho et al., 2018; Almiñana and Bauersachs, 2019). In addition to other molecules, maternal EVs (e.g. from oviduct cells or follicular fluid) improve embryo development, morphological quality, blastocysts rate, hatching ability, total cell number and cryotolerance (Lopera-Vásquez et al., 2016, 2017). Embryo-derived EVs also interact with the maternal side via their cargo (Pap et al., 2008; Atay et al., 2011; Pallinger et al., 2018; Ávila and Silveira, 2019). Likewise, EVs secreted by early embryos also participate in embryonic crosstalk during *in vitro* culture (Saadeldin et al., 2014; Qu et al., 2017; Pavani et al., 2018; Kim et al., 2019).

The concentration, size distribution and cargo of EVs, are modulated by cell function and different stimuli (Colombo et al., 2014). This information led to the use of EVs as markers for the diagnostics of different pathologies (Lane et al., 2018). Pre-implantation embryos also release different populations of EVs according to their intrinsic quality and culture conditions (Tannetta et al., 2014; Mellisho et al., 2017, 2019). These facts constitute the conceptual foundation for using embryo derived EVs as non-invasive markers to select *in vitro* produced embryos, with higher developmental competence (Mellisho et al., 2017, 2019). The proposed approach includes EVs separation (colloquially referred as isolation) from embryo culture medium and their characterization. However, natural derived protein sources commonly used to support embryo development, such as fetal bovine serum (FBS) and bovine or human serum albumin (BSA, HSA) can contribute to the population of nanoparticles in culture medium. These nanoparticles could include high amount of EVs, which are undistinguished from those secreted by the embryo (Théry et al., 2006; Eitan et al., 2015; Mellisho et al., 2017; Liao et al., 2017; Mellisho et al., 2019). One alternative to avoid the bias of non-embryonic EVs is removing protein source from culture medium. However, protein supplementation is required to support embryo development during *in vitro* culture (Li et al., 2015). Pavani et al. (2018) reported that culture medium supplemented with BSA contain nanoparticles but no EVs. However, other research showed that BSA and HSA contains vesicles that may affect cells in culture (Witwer et al., 2013; Shelke et al., 2014; Stolk and Seifert, 2015).

An accepted alternative is the use of EVs/nanoparticles-depleted medium. This can be achieved by depleting the final, ready-to-use culture medium or particularly the protein source. Several protocols have been proposed for this purpose (Théry et al., 2006; Lehrich et al., 2018; Guerreiro et al., 2018). However, several works showed that EVs/nanoparticles depletion reduced the beneficial effects of bovine and human serum on growth and survival of cultured cells (Eitan et al., 2015; Lehrich et al., 2018). This could be probably to the fact that EVs from protein source can be internalized by cells in culture, therefore, they can alter cellular functionality (Cahoy et al., 2008; Zhang et al., 2014; Eitan et al., 2015).

Earlier we separated and identified extracellular vesicles secreted by bovine *in vitro* cultured embryos during hatching (Mellisho et al., 2017). In that work, blastocysts were cultured individually in EVs-depleted medium from day 7-9 without negative effect on embryo viability. However, when embryos were selected at morula stage and cultured individually in EVs-depleted medium, blastocyst rate was decreased (Mellisho et al., 2019). This could be attributed to the absence of EVs from the culture medium, the loss of embryo derived EVs or a combination of both. Based on that, the aims of this study were to (1) evaluate if the culture medium subjected to nanoparticle depletion can support the proper *in vitro* development bovine embryo and (2) analyze whether embryos are able to internalize culture medium nanoparticles at different developmental stages.

## Methods

All experiments were approved by the Ethics Committee of the Faculty of Veterinary Sciences, Universidad de Concepción under the permission number CBE-27-2019.

### Experimental design

**Figure 1:**
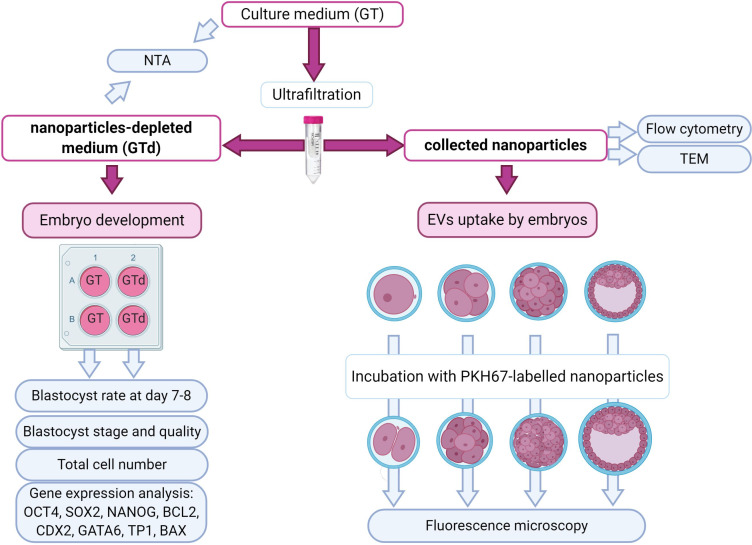
Experimental design. Culture medium (Global Total: GT) was depleted of nanoparticles by ultrafiltration. The depleted culture medium (GTd) was used for embryo culture in both experiments. GT and GTd were analyzed by nanoparticles tracking analysis (NTA). Separated nanoparticles from GT were additionally analyzed by flow cytometry for EVs surface markers and by transmission electron microscopy (TEM). Zygotes were cultured on groups on GTd or GT for embryo development assessment at day 7 and 8 post-IVF. Nanoparticles from GT were labelled and added to embryo culture in GTd for EVs uptake evaluation by fluorescence microscopy, at different developmental stages.

To accomplish the proposed goals, two experiments were performed. In the first experiment, commercial culture medium Global Total (GT) that contains human derived protein supplement (HSA- 4.4 mg/mL and α- and β-globulins- 0.6 mg/ml; LGGT-030, Lot number LGGT-170602C; CooperSurgical LifeGlobal, Guilford, CT, USA) was nanoparticle depleted; both depleted medium and nanoparticles were collected. The effect of culture medium depletion on pre-implantation development of bovine embryos produced by *in vitro* fertilization (IVF) was evaluated. After IVF, presumed zygotes were randomly assigned to groups of 25-30 to depleted culture medium (GTd) or to control non-depleted medium (GT). Embryos were cultured in controlled atmosphere for 8 days. Blastocysts rate was determined at day 7 and 8 of development. Blastocysts quality was assessed by their morphology, total cell count and gene expression of key developmental candidate genes (Figure 1 left panel).

Then, nanoparticles isolated from culture medium were characterized by their morphology, size distribution and concentration, and the presence of EVs markers. In the second experiment, nanoparticles were labeled with fluorescent dye (PKH67) to assess whether preimplantation bovine embryos internalize those particles.

Stained nanoparticles were added to embryo culture medium in a similar quantity as their normal amount in non-depleted medium (calculated by NTA). For this, the concentration of particles in total culture medium (before depletion) was determined and added the same number of labeled particles to depleted medium. Embryos at different developmental stages after IVF (Days 1, 2.5, 4 and 6; IVF=Day 0) were incubated for 24 h in medium with labelled nanoparticles (Figure 1 right panel). The presence of particles inside embryonic cells was evaluated in a fluorescent inverted microscope.

### *In vitro* embryo production

Ovaries from beef cattle were collected at a local abattoir (Frigosur Ltda, Chillan, XVI Region – Ñuble), following standard procedures described by Velasquez et al. (2016). The cumulus oocyte complexes (COCs) were collected and *in vitro* matured (IVM) for 20-22 h in four-well dishes (25 to 30 COCs per 500µL well) in TCM-199 supplemented with follicle stimulating and luteinizing hormones (0.01 U/ml each), 17β-estradiol (1μg/ml), epidermal growth factor (EGF; 10ng/ml), and 10% FBS at 39 °C in a 5% CO2 in air atmosphere. Oocytes were fertilized using frozen–thawed commercial semen from a single bull of proven fertility (Semex, Madison, WI, USA). IVF was performed following standard protocols used in our laboratory (Velásquez et al., 2016). After 18-20 h of IVF, cumulus cells were mechanically removed by 4 min vortexing in TCM-Hepes with 0.3 mg/ml of hyaluronidase. Thereafter, embryos were washed three times in depleted medium before culture. Presumptive zygotes were *in vitro* cultured (IVC) in groups on four-well plates (25 to 30 zygotes per 500 µL well) in GT or GTd medium according to the experimental group. Culture dishes were placed in an atmosphere containing 5% CO_2_, 5% O_2_ and 90% N_2_, 100% humidity at 39°C until day 8.

### Characterization of nanoparticles and depleted medium

According to a previous analysis Nanoparticles Tracking Analysis carried out in our laboratory, ultracentrifugation-based protocol (100000 xg, 18 hr) depleted 34% of nanoparticles from complete culture medium in comparison to 74% obtained with an ultrafiltration-based protocol. Because of that, in this study nanoparticles depleted culture medium (GTd) was produced by ultrafiltration (centrifugal filter devices 100 kDa, Amicon, Merck, Darmstadt, Germany) of complete medium for 15 minutes at 1660 x g at 4°C. Both, supernatant (depleted medium) and pellet (nanoparticles) were collected and stored at -80°C.

### Nanoparticles Tracking Analysis (NTA)

Culture medium (GT: 500 μL), nanoparticles depleted culture medium (GTd: 500 μL), isolated nanoparticles (10 μL nanoparticles-suspended pellet in 500 μL of PBS), PBS (as negative control) and bovine follicular fluid (FF; as positive control) were subjected to nanoparticles tracking analysis (NTA) on a NanoSight NS300 (Malvern Instruments Ltd, Malvern, UK) equipped with a 488 nm and a high-sensitivity sCMOS camera, to determine the presence, concentration and size distribution of nanoparticles. Additionally, samples were immunolabeled by incubating with primary antibodies against CD9 (FITC-conjugated; catalog no. 34162, Abcam, Cambridge, U). This was performed to determine the percentage of total nanoparticles positive to a classic EV marker. Immunolabeled samples were subjected to NTA using a 488 nm laser and a 500 nm filter for fluorescence detection.

Data were determined at 20 to 100 particles per frame. Negative control (PBS) had less than one particle per frame. Samples were injected at room temperature (RT) in a continuous flow with a syringe pump into the sample chamber. Analysis of each sample was performed as described by Mellisho et al. (2017). Graphical analysis showed particle size distribution of the nanoparticles in the groups and concentration was reported as particles per milliliter.

### Transmission Electron Microscopy analysis (TEM)

Transmission electron microscopy (TEM) was used to identify the morphology of nanoparticles detected by NTA following the protocol described by Théry et al. (2006). In brief, the nanoparticles-suspended pellet (10 μL) was deposited on formvar-carbon-coated copper grids and let it dry at room temperature. Samples were contrasted first in a solution of uranyl oxalate, pH 7 and then contrasted and embedded in a mixture of 4% uranyl acetate and 2% methyl cellulose in a ratio of 100 μL/900 μL, respectively. Grids were visualized in Unidad de Microscopía Avanzada UC (UMA UC) on a Philips Tecnai 12 transmission electron microscope, operated at 80 kV and the images were processed using iTEM software.

### Flow cytometry

Finally, the phenotype of nanoparticles was evaluated by identification of EVs surface markers using flow cytometry following the protocol described by Théry et al. (2006) with modifications. Nanoparticles (35 μL or 4 x 10^8^ particles/ml) were incubated with 4 μm aldehyde/sulfate latex beads (0.125 μL or 1.25 x 10^5^ particles/ml) (ThermoFischer Scientific, Santiago, Chile). The nanoparticles/beads complexes were incubated with primary antibodies against CD63 (FITC-conjugated; catalog no. 18235, Abcam, Cambridge, UK), CD9 (FITC-conjugated; catalog no. 34162, Abcam, Cambridge, UK), CD81 (PE-conjugated; catalog no. 81436, Abcam, Cambridge, UK) or CD40L (PE/Cy5®-conjugated; catalog no. 25044, Abcam, Cambridge, UK) for 2 h at 4°C. Nanoparticles/EVs separated from bovine follicular fluid and from human cells culture supernatant were used as positive controls (Mellisho et al., 2017). A negative control antibody reaction was performed using latex beads alone incubated with each antibody for 2 hours at 4°C. The labelled Nanoparticles/beads complex were resuspended in focusing fluid and subjected to flow cytometry using Attune™ NxT Flow Cytometer (ThermoFischer Scientific, Santiago, Chile).

### Assessment of embryo quality and blastocyst rate

Blastocyst rate was calculated as the total number of blastocysts among the total presumptive zygotes per experimental group at day 7 and 8 after *in vitro* fertilization. Blastocysts were classified morphologically following the criteria of the International Embryo Technology Society (IETS) to define blastocyst stage (blastocyst: 6, expanded blastocyst: 7, hatching blastocyst: 8 and hatched blastocyst: 9) and quality (excellent or good: 1, fair: 2 and poor: 3) (Bo and Mapletoft, 2013). Day 8 blastocysts were selected for total cell number (n=14) or gene expression analysis (n=50). For cell count, individual embryos were incubated for 10 minutes on Hoechst 33342 (NucBlue® ReadyProbes®) at 10% for nuclei dye and observed on fluorescence microscopy EVOS FL Imaging System (ThermoFischer Scientific, Santiago, Chile).

### Gene expression analysis

The expression analysis of developmental crucial genes (NANOG, SOX2, OCT4, CDX2, GATA6, TP1, BAX and BCL2L1) was performed by real-time PCR. Five pools of 5 blastocysts each (quality 1 and 2), were organized per experimental group (GT and GTd). Embryos were frozen and stored at -80 °C until analysis. RNA extraction was performed by adding 50 µL of lysis buffer from Cells-to-cDNA TM II kit (Ambion Co., Austin, TX, USA). All samples were treated with DNase I (0.04 U/mL) for genomic DNA digestion. Total RNA was not quantified and hence, 7 µL of total RNA was used for cDNA conversion using SuperScript® IV First-Strand kit and following manufacturer's instruction (Thermo Fisher, Santiago, Chile). The cDNAs were kept frozen at -20 °C until PCR.

Gene expression level was performed by real-time PCR using the ∆∆Ct method. Primer efficiency was evaluated using a standard curve and were used those with efficiency range of 90–110% and a correlation coefficient of at least 0.9. PCR reaction was performed in a final volume of 10 µL with 2 µL of cDNA from each sample, 1 µL of primers (10 pmol each, forward and reverse) and 5 µL of 2x Sensimix SYBR Hi-ROX (Bioline, Berlin, Germany) and run on a MX3000P Real-Time PCR device (Agilent, Santa Clara, CA, USA). All samples were loaded as duplicates (technical replicates). Melting curves and Ct values were calculated with built-in software. The expression level of each gene was normalized using ACTB as housekeeping gene (Velásquez et al., 2019). Relative expression levels between groups (GTd and GT) were expressed as 2^-∆∆Ct^. Primers used and PCR conditions for each gene are presented in Table 1.

**Table 1:** Oligonucleotide composition and PCR conditions for the analyzed genes from bovine embryos.

**Gene**	**Primer sequences**	**Accession N°**
ACTB	F: 5´ - GGCCAACCGTGAGAAGATGACC 3´	BT030480.1
	R: 5´ - GAGGCATACAGGGACAGCACAG 3´	
OCT4	F: 5´ - GGAGAGCATGTTCCTGCAGTGC 3´	NM_174580
	R: 5´ - ACACTCGGACCACGTCCTTCTC 3´	
SOX2	F: 5´ -CGAGTGGAAACTTTTGTCCG 3´	NM_001105463
	R: 5´ -GGTATTTATAATCCGGGTGTT 3´	
NANOG	F: 5´ - TTCCCTCCTCCATGGATCTG 3´	NM_001025344
	R: 5´ - ATTTGCTGGAGACTGAGGTA 3´	
BAX	F: 5´ -AGGGTTTCATCCAGGATCGAGC 3´	NM_173894.1
	R: 5´ - TCATCTCCGATGCGCTTCAGAC 3´	
BCL2L1	F: 5´ - GAAAGCGTAGACAAGGAGATG 3´	NM_001077486.2
	R: 5´ - CCGTAGAGTTCCACAAAAGTG 3´	
TP1	F: 5´ - GCCCTGGTGCTGGTCAGCTA 3´	NM_001015511.4
	R: 5´ - CATCTTAGTCAGCGAGAGTC 3´	
CDX2	F: 5´ - CCTGTGCGAGTGGATGCGGAAG 3´	NM_001206299.1
	R: 5´ - CCTTTGCTCTGCGGTTCT 3´	
GATA6	F: 5´ - CCAGAATTTCTCCGCCCCTT 3´	XM_002697727.3
	R: 5´ - CGGAGGTGTGTACCAAACGA 3´	

### Nanoparticles labeling and uptake

In the second experiment, the recovered nanoparticles (200 µL) were obtained by ultrafiltration of the culture medium (as described before) and labeled with PKH67 (Sigma-Aldrich, St. Louis, MO, USA) according to manufacturer’s protocol for exosome labeling. The recovered nanoparticles were washed twice with PBS. For the treatment of embryos at each developmental stage with labelled nanoparticles, 25 µL of culture medium-derived nanoparticles were diluted in 300 µL of diluent C. Then, 2 µL of PKH67 was added to diluent C mixed with nanoparticles and held at room temperature for 5 minutes in the dark. Dye was quenched by adding 10% BSA in PBS and labelled nanoparticles were washed 3 times by ultrafiltration (0.5 mL centrifugal filter device 100 kDa, Amicon, Merck, Darmstadt, Germany) for 20 minutes at 2500 x g. For negative control, sterile PBS was incubated with PKH67 and treated in the same manner as described above. The labeled nanoparticles (treatment) or PBS (negative control) were added to 500 µL of depleted medium (GTd).

Embryos were produced following standard protocol described before, cultured in groups in control medium (GT). Embryos were classified at days 1, 2.5, 4 and 6 after IVF and placed in depleted medium (500 µL per well) supplemented with labeled nanoparticles (3×10^9^ particles per 500µL) or PBS during 24 hrs. After the given time points, 2-cell embryos, 8-cells embryos, morulae and blastocysts respectively were incubated for 10 minutes on 10% Hoechst 33342 (NucBlue®️ ReadyProbes®️) for nuclei staining, on a coverslip and photographed on fluorescence microscopy EVOS FL Imaging System to ascertain the presence of fluorescent labeled nanoparticles inside the blastomeres. The experiment was repeated three times what means that three independent groups of embryos were produced.

### Statistical analysis

First, Shapiro-Wilk test was performed to all variables to examine its normality. Concentration of nanoparticles present in GT and GTd was compared with student’s t-test (two tails) as well as the percentage of blastocyst obtained on day 7 and 8, percentage of blastocyst stages at day 7, total cell count and diameter of blastocysts. Mean and mode size of nanoparticles, percentage of blastocyst stages at day 8, and levels of gene expression were carried out through a nonparametric U Mann-Whitney test. In all cases the differences were considered significant with a value of P <0.05. All analyzes were carried out with the statistical program InfoStat (Buenos Aires, Argentina, 2002).

## Results

### Characterization of nanoparticles from culture medium

**Figure 2:**
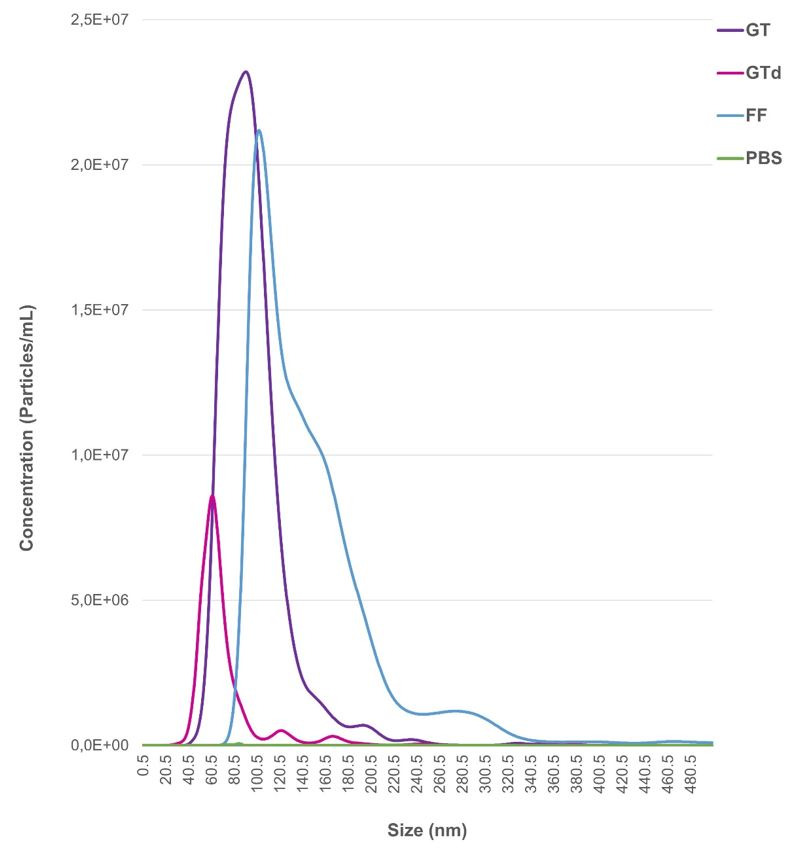
Nanoparticles tracking analysis of culture medium used for embryo culture. The horizontal bars indicate particle size range while the y axis indicates concentration of nanoparticles. GT: Non depleted medium; GTd: depleted medium (GT); PBS: negative control; FF: EVs separated from bovine follicular fluid used as positive control.

Embryo culture medium (GT) was depleted from nanoparticles (GTd). Both, GT and GTd were analyzed by NTA to determine the presence and characteristics of particles (mean size and mode and concentration; Table 2 and Figure 2). The protocol used to deplete the culture medium eliminated almost 90% the nanoparticles (Table 2, Figure 2). The separated GT nanoparticles that were incubated with antibody against CD9, had 17.2% fluorescence positiveness. However, 0.13% of nanoparticles detected in depleted medium were fluorescent and consequently positive to CD9.

**Table 2:** Measurements of the size and concentration of nanoparticles separated from culture medium used for embryo culture, according to the nanoparticles tracking analysis.

**Sample**	**Size (mean) (nm)**	**Size (mode) (nm)**	**Particles concentration (particles/mL)**
GT	97.6 ± 2.2	83.1 ± 5.3	1.27x10^9^ ± 7.96x10^7^
GTd	73.3± 3.4	58.8 ± 2.9	2.45x10^8^±4.35x10^6^
FF	148.6 ± 0.3	102.7 ± 2.2	1.50x10^9^ ± 6.15x10^7^
PBS	111.5 ± 56	53.5 ± 26.9	1.73x10^6^ ± 9.23x10^5^

GT: non-depleted culture medium; GTd: depleted culture medium. FF: EVs separated from bovine follicular fluid used as positive control; PBS: negative control.

**Figure 3:**
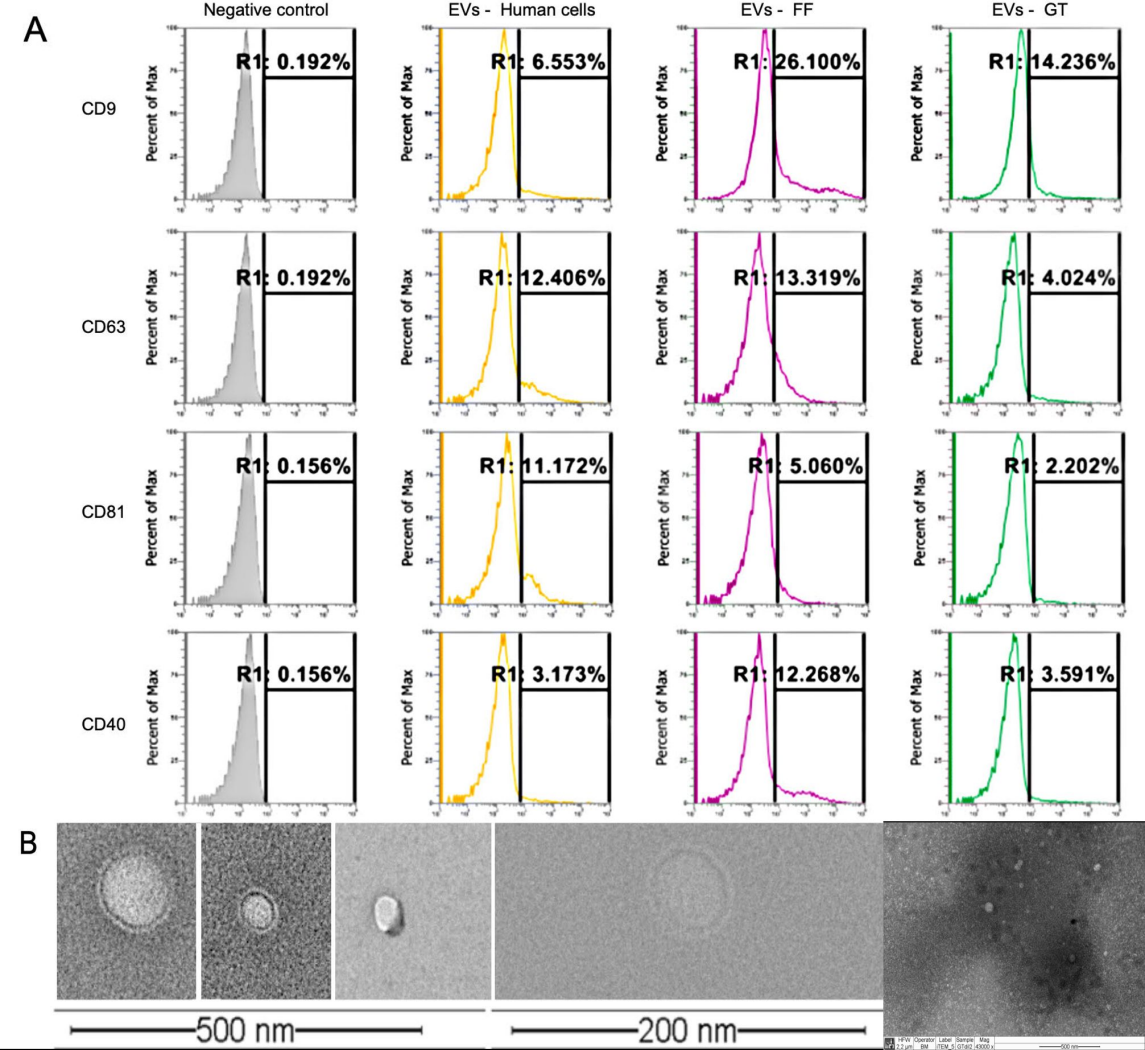
Characterization of nanoparticles separated from culture medium. (A) Flow cytometry analysis of EVs markers (CD9, CD63, CD81 and CD40; EVs-GT: particles separated from embryo culture medium. EVs-human cells and EVs-FF are particles separated from culture medium of human cells and bovine follicular fluid respectively, used as positive controls. Negative Controls: beads without EVs and incubated with Ab; (B) Representative images from transmission electron micrographs showing nanoparticles present in culture medium.

Particles separated from culture medium were visualized by TEM but very few showed a classical EVs morphology. However, recovered nanoparticles and positive controls (EVs from bovine follicular fluid and from human cells culture medium supernatant) were positive to EVs specific surface markers CD9, CD81, CD63 and CD40L (Figure 3) whereas negative control showed 0.1% positivity for all markers (Figure 3A).

### Embryo development on nanoparticles-depleted medium

**Figure 4:**
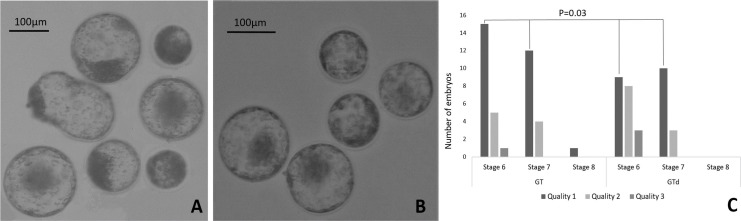
Morphological classification of Day 8 blastocysts. Representative picture of Day 8 blastocysts derived from in vitro produced bovine embryos and cultured in non-depleted (GT; A) or in depleted medium (GTd; B); (C) Number of embryos in each category considering blastocyst stage and quality accordingly to IETS manual.

**Figure 5:**
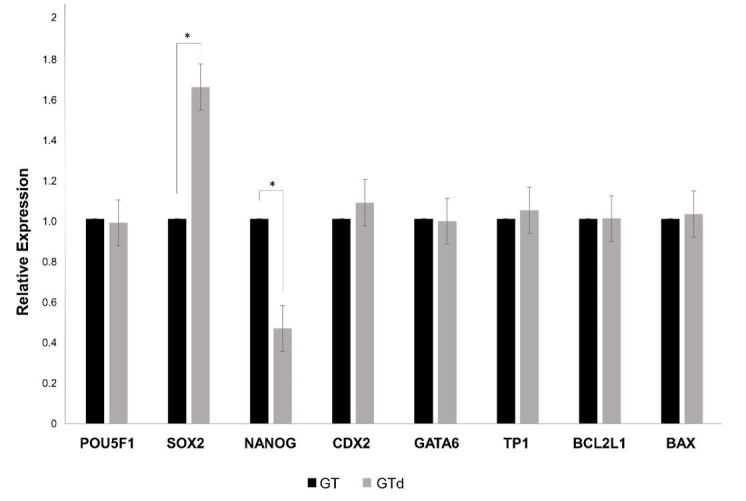
Gene expression analysis in blastocysts derived from in vitro-produced bovine embryos cultured in depleted medium (GTd) and non-depleted medium (GT). The expression level of each gene was normalized to the expression of ACTB. *indicates statistical differences between groups (P < 0.05).

To assess the effect of depletion on pre-implantation development, *in vitro* produced embryos were cultured in nanoparticles-depleted medium or in non-depleted medium (control). Nanoparticles depletion did not affect blastocyst rates at Day 7 and Day 8, total cell number neither blastocyst diameter (Table 3). However, the number of blastocysts of excellent or good quality (1), judged by the morphology according the IETS criteria [58], was significantly higher in embryos cultured in non-depleted medium, but less advanced in development at day 8 in comparison to embryos cultured in depleted medium (p=0.03) (Figure 4). On the other hand, the expression level of SOX2 was significantly higher in blastocysts cultured in depleted medium whereas NANOG expression was significantly lower in these embryos (Figure 5). The other genes were equally expressed in blastocysts from both groups.

**Table 3:** Effect of nanoparticle-depleted medium on in vitro bovine embryo development.

**Culture medium condition**	**No. zygotes**	**D7 blastocyst (%)**	**D8 blastocyst**		
			**No. (%)**	**No total cells (±SD)**	**Diameter (µm) (±SD)**
GT	149	34 (22.8)	38 (25.5)	99.8 (±41.8)	191 (±26.2)
GTd	151	23 (15.2)	33 (21.8)	100 (±14.9)	176.6 (±19.4)

GT: non-depleted culture medium; GTd: depleted culture medium. D7, D8: days of embryo development after in vitro fertilization (D0). SD: standard deviation.

### Uptake of culture medium-derived nanoparticle by early embryos

**Figure 6:**
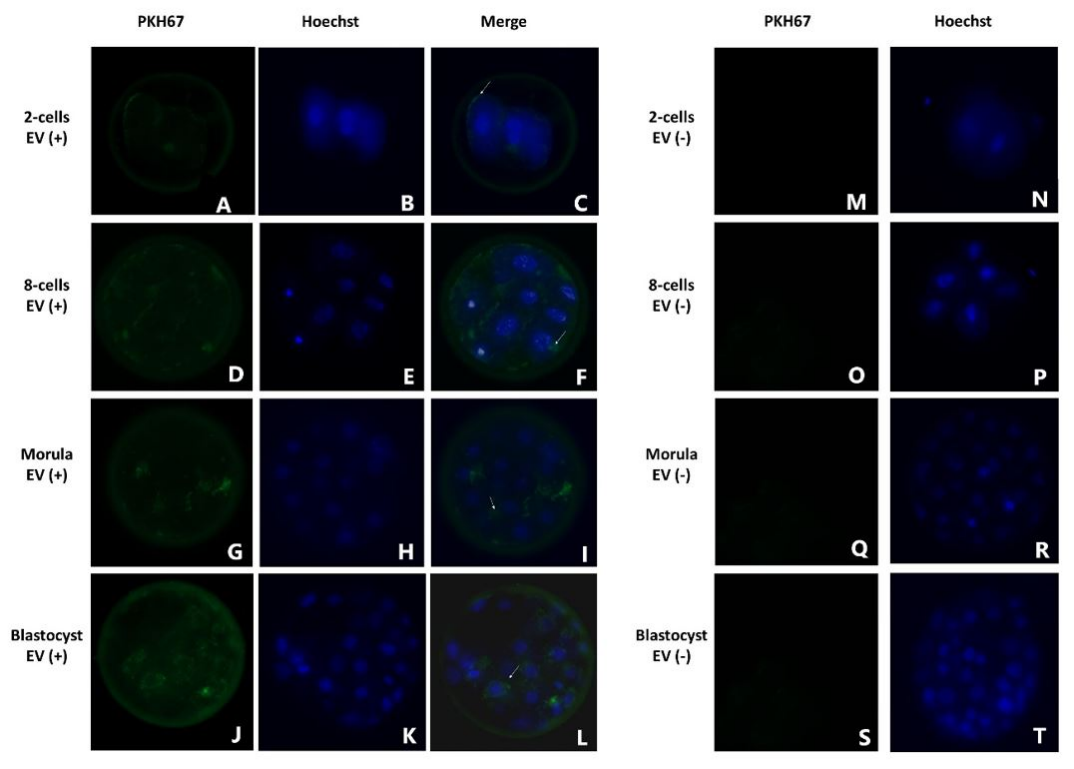
Fluorescence images of bovine embryos at different developmental stages showing internalization of nanoparticles derived from culture medium. Nanoparticles from GT medium were stained with PKH67 dye and added to depleted culture medium. Embryos were incubated with stained nanoparticles for 24 hours. Images are representative of each stage (A-C: 2-cells embryo; D-F: 8-cells embryos; G-I: morula; J-L: blastocysts; M-T: embryos after incubation with negative control (PKH67-PBS). Arrows highlight nanoparticles that passed though the zona pellucida and are internalized by embryonic cells.

Embryos at different stages were incubated during 24h with PHK67-stained nanoparticles recovered from GT medium. After incubation, the presence of stained nanoparticles within embryonic cells in 2-cells embryos, 8-cells embryos, morulae and blastocysts was evaluated in a fluorescent inverted microscope. Labeled nanoparticles were observed in the perivitelline space as well as in blastomeres in all evaluated stages (Figure 6). However, in two-cell embryos, the fluorescent particles were localized close to the cell membrane (Figure 6C) while in other embryonic stages, particles were spread throughout the cytoplasm, around the nucleus (Figure6F, I and L). No fluorescence was observed when embryos were incubated with PHK67-labeled PBS (negative control; Figure 6MT).

## Discussion

The characteristics of nanoparticles secreted by embryos produced *in vitro*, have become attractive to design new strategies for selecting those with greater chance to produce a healthy offspring. Current separation methods do not discriminate EVs secreted by the embryos from those present in culture medium. To avoid any quantitative and/or qualitative assessment of EVs derived from commercial either handmade medium, it has been utilized depletion methods to eliminate the significant number of EVs found in complete culture medium (Lehrich et al., 2018). However, EVs-depleted culture medium can reduce growth and alter the phenotype of *in vitro* cultured cells (Brunner et al., 2010; Aswad et al., 2016; Lehrich et al., 2018). Previously, we observed that individual culture of bovine embryos from morula to blastocysts stage, in EVs-depleted medium, reduced blastocyst rate at day 7, from 30 to 12% (Mellisho et al., 2017).

In this work commercial culture medium Global Total (GT) which contains human-derived proteins (serum albumin and β-globulins) was used for embryo culture. The presence of EVs in culture medium supplemented with serum albumin (human-HSA or bovine-BSA) has been widely discussed. Pavani et al. (2018) reported that culture medium supplemented with BSA does not contain nanoparticles fulfilling EVs characteristics (morphology and presence of surface markers). However other authors describe the presence of EVs in culture medium conditioned with serum albumin (Witwer et al., 2013; Shelke et al., 2014; Stolk and Seifert, 2015). Several authors describe that BSA and HSA-derived EVs have a large amount of RNA, DNA and protein and have a functional effect protecting cultured cell from starvation-induced apoptosis (Stolk and Seifert, 2015; Hammond et al., 2017).

The culture medium was depleted of EVs/nanoparticles by ultrafiltration using the Amicon ® 100 kDa centrifugal filter devices, a protocol standardized in our laboratory, which allow the albumin (66.5 kDa) to pass through the filter, concentrating nanoparticles in the filter device. We found that this protocol reduced nearly 90% of nanoparticles concordant with results from Lehrich et al. (2018). Kornilov et al. (2018) also demonstrated that ultrafiltration-based protocol depleted EVs from FBS more efficient than ultracentrifugation and commercial methods. By TEM, only few nanoparticles collected from culture medium showed an EV morphology with the presence of membrane. However, small nanoparticles without an external membrane were identified. Similar particles have been described as exomeres that are nanoparticles also secreted by cells with a functional cargo and that are internalized by cell in culture (Zhang et al., 2018; Zhang e al., 2019). These nanoparticles also share molecular markers with EVs such as CD81 and CD9 (Zhang et al., 2019). In this work, collected nanoparticles were positive to EVs markers. A low population of the nanoparticle/bead complex was positive for each marker in the culture medium samples; however, this was similar in the positive controls and in embryo-derived EVs as previously reported (Mellisho et al., 2017). This could be explained by a low proportion of nanoparticles/beads.

Nanoparticles-depletion protocol used did not affected blastocyst rate, total cell number neither blastocyst diameter in cultured bovine embryos. In this study, embryos were cultured in groups during the entire period, consequently, the beneficial effect of embryo-derived EVs and embryo-embryo paracrine signaling is maintained, probably counteracting the lack of nanoparticles from the medium. The depleted medium still had a population of small particles, but the largest nanoparticles were removed with the used protocol. Those particles might be supporting embryo development in line with the report from Lee et al. (2019). These authors showed that a fraction of EVs, smaller than exosomes, contain factors that stimulate cell proliferation (Lee et al., 2019).

The internalization of nanoparticles derived from culture medium by bovine embryos in different developmental stages was proven; stained particles were observed in the cytoplasm surrounding the nucleus from 2-cells stage and on. Nanoparticles separated from culture medium had a diameter < 100 nm therefore, it is not surprising that they can pass through the zona pellucida of the bovine embryos considering that the pores size is 155 and 223 nm depending on embryo stage (Vanroose et al., 2000). Several reports have demonstrated that pre-implantation embryos uptake EVs from different origin and that those EVs may impact on embryo development and expression of genes in the embryo (Saadeldin et al., 2014; Almiñana et al., 2017; Pavani et al., 2018).

The cargo of nanoparticles derived from culture medium was not evaluated in this work but the change in the expression of pluripotency markers in the blastocysts, suggests that they could carry molecules able to modify the functionality of the embryos. The population of nanoparticles from culture medium might have a benefit effect on embryo quality, jugged by the lower expression of SOX2 and higher expression of NANOG. It is known that pluripotency markers OCT4, SOX2 and NANOG are essential for normal development of bovine embryo beyond blastocyst stage (Beyhan et al., 2007; Rodríguez-Alvarez et al., 2013). However, in a previous work, our group demonstrated that the expression level of SOX2 at blastocyst stage negatively correlates with further embryonic development (Velásquez et al., 2019). In concordance with this, Pan and Schultz (2011) reported that over expression of SOX2 due to injection of complementary RNA in one-cell mouse embryos, induces developmental arrest of the embryos at two cells stage. On the other hand, NANOG expression is required to maintain the epiblast cell in bovine embryos; hence, its down regulation will affect further embryo development (Simmet et al., 2018). The higher expression of NANOG and lower expression of SOX2 in embryos cultured in non-depleted culture medium might be due to the presence of molecules, such as microRNAs carried by nanoparticles internalized by blastomeres. For instance, Wang et al. (2017) reported that the microRNA-148a improves the development of porcine embryos by reducing DNA methylation and increasing expression of OCT4 and NANOG. In human amniotic epithelial stem cell, microRNA-145 inhibits SOX2 expression, weakening their pluripotency (Zou et al., 2016).

## Conclusion

From the results of this work, we conclude that medium used for embryo culture is a source of EVs and/or exomeres-like nanoparticles which pass through the zona pellucida and are internalized by embryonic cells. These nanoparticles, potentially modifying gene expression of, for instances, pluripotency genes. Without the definition of the actual cargo, it is difficult to speculate about the mechanisms governing the changes in gene expression in the embryos. Another possibility is that not only the absence of those nanoparticles, but also depletion of certain proteins not necessarily engulfed in EVs could play a yet unknown role in embryo development. We were unable to find data in literature regarding the loss of specific molecules (e.g., proteins) discarded along with nanoparticles depletion procedures, though this concern has been raised by others (Pavani et al., 2018). Based on that, it will be of great interest to investigate how the composition of the medium or of its protein source like FBS and serum albumin is modified after EVs-depletion protocols. This could help in optimizing and tailoring culture media for the study of EVs secreted by cells or embryos.
